# Antischistosomal and anti-inflammatory activity of garlic and allicin compared with that of praziquantel in vivo

**DOI:** 10.1186/s12906-018-2191-z

**Published:** 2018-04-27

**Authors:** Dina M. Metwally, Ebtesam M. Al-Olayan, Mohammad Alanazi, Sanaa B. Alzahrany, Abdelhabib Semlali

**Affiliations:** 1 Zoology Department, Faculty of Science, King Saud University, P.O. Box 2454, Riyadh, 11451 Kingdom of Saudi Arabia; 2 Parasitology Department, Faculty of Veterinary Medicine, Zagazig University, Zagazig, Egypt; 3 Department of Biochemistry, College of Science, King Saud University, Riyadh, Kingdom of Saudi Arabia

**Keywords:** Allicin, *Schistosoma mansoni*, Proinflammatory cytokines

## Abstract

**Background:**

Schistosomiasis is an acute and chronic zoonotic parasitic disease caused by trematode worms. The host inflammatory response to schistosome eggs leads to perioval granulomata formation, mainly in the liver and intestine. This study investigated the potential antischistosomal and anti-inflammatory activity of both garlic extract and allicin on liver fibrotic markers in BALB/c mice with schistosomiasis (*S. mansoni* infection) compared with that of the commonly used drug, praziquantel (PZQ).

**Methods:**

In this study, 140 female BALB/c mice (7-weeks old) were divided into seven groups with 20 mice each. Six groups were infected with *S. mansoni* cercariae and treated with garlic, allicin, or PZQ. The seventh group was the negative control. Twenty-four hours after the final treatment, the mice were euthanised and perfused for worm recovery. The liver and intestines were harvested for parasitological and histological assessment and to analyse the proinflammatory cytokine mRNA expression.

**Results:**

Prophylactic administration of garlic and allicin to the infected mice significantly reduced the worm burden. Serum concentrations of liver fibrosis markers and proinflammatory cytokines were also reduced. PZQ was the most efficacious for reduction in the number of worms. These results are similar to those normally obtained using PZQ.

**Conclusions:**

Crushed garlic homogenate and allicin are potential complementary treatments that may be used with PZQ.

## Background

Schistosomiasis is one of 17 top priority neglected tropical diseases recognised by the World Health Organization [1]. It is caused by blood flukes belonging to the genus *Schistosoma* and is a chronic illness common in humans living in underdeveloped tropical and subtropical countries in Africa, Asia, the Caribbean, and South America [2]. Seventy-eight countries are considered endemic for schistosomiasis, and 258 million people required preventive chemotherapy in 2014. This prevalence may be repeatedly noted in future [3]. Due to the prevalence of variations in the intermediate snail host species, patterns of water exposure, and other sociocultural factors, schistosomiasis is not uniformly distributed in endemic areas [4]. The major schistosome species infecting humans are *S. haematobium*, *S. mansoni*, and *S. japonicum. S. mansoni* lives in intestinal venules and primarily affects the liver and gut [5, 6]. The disease results from the eggs of small, thread-like parasitic worms living in the blood vessels of the liver, gut, and bladder [7]. The host tissue inflammatory response to the schistosome eggs leads to perioval granulomata formation, especially in the liver and intestines. In the liver, infection results in chronic portal fibrosis. Fibrotic tissue is composed of extracellular matrix (ECM) and connective tissue cells. Collagen types 1, 3, 4, and 5; type 3 procollagen; fibronectin; and laminin are major components of fibrous tissue in the liver present due to *S. mansoni* schistosomiasis infestation [8]. Fibroblasts are the most important connective tissue cells for ECM production in normal livers. In response to injury, fibroblasts and smooth muscle cells proliferate and produce an extensive collagen network [9]. Treating infected individuals with the anthelminthic drug praziquantel (PZQ) controls infection and morbidity [10, 11]. PZQ is safe, broadly therapeutic, and inexpensive. Relying on a single drug to treat schistosomiasis can lead to possible drug resistance [12, 13]. Therefore, the improvement and advancement of potential alternatives for controlling schistosomiasis has been delayed [14]. Both garlic and *Nigella sativa* have promising antischistosomal activities [15]. Reduced *S. mansoni* worm burden and egg counts, normalised liver enzymes, and improved antioxidant status are noted after both garlic and onion oil treatment [16]. According to Mehlhorn et al. [17], garlic is also described as an anthelmintic agent. However, its efficacy against endoparasites may be associated with the action of herbal plant agents or the stimulation of a high rate of food passage into the gastrointestinal tract cause by the oil contained in this phytotherapy. This study evaluated the potential antischistosomal and anti-inflammatory activity of raw garlic and the garlic supplement allicin in a trial to determine its effectiveness compared to PZQ in reducing pathological changes caused by *S. mansoni* infections.

## Methods

### Garlic homogenate preparation

Fresh garlic was purchased at markets in different regions of Cairo, Egypt. Garlic bulbs were separated, peeled, and washed with distilled water. After drying in a shed, 500 g of clean garlic bulbs were crushed using a commercial blender (Braun, Germany). The resulting paste was diluted with distilled water (1 g/mL) to prepare an aqueous solution. The solution was filtered properly (0.45 μm) to remove such ambiguity. Raw garlic juice was placed in 1.5 mL tubes and stocked in a −20°C freezer. A working solution (50 mg/kg body weight) was prepared from the stock solution by diluting it with distilled water. The selected dose for the present study (50 mg/kg body weight) corresponds to the daily amount of garlic recommended for human consumption (4 mg) [18]. The garlic extract was administered to laboratory animals by oral gavage. Allicin was obtained in liquid form (1000 ppm) from Allicin International, Ltd. (Rye, East Sussex,UK). The substances were stored at 4 °C and were retrieved only during use.

### Animals and experimental design

One hundred forty 7-week-old female BALB/c mice (25–30 g) were obtained from the experimental research center of Theodor Bilharz Institute, Cairo, Egypt. The animals were kept in wire-bottomed cages in a room under standard conditions with a 12-h light-dark cycle at 25°C ± 1 °C for 1 week until treatment. The mice were provided tap water and a balanced diet ad libitum. Cercariae, from an Egyptian strain of *S. mansoni,* were isolated from laboratory-raised infected *Biomphalaria glabrata* snails [19]. Cercariae shedding was induced by exposing infected water-immersed snails to light for 1.5 h. The cercariae were collected by cooling and low-speed centrifugation. After collection from the infected snails, the cercariae were placed on cover slips and counted under a dissecting microscope. The mice were infected percutaneously with approximately 100 ± 2 *S. mansoni* cercariae by the paddling method [20]. In brief, the mice were individually placed into 600-mL plastic beakers containing 80 mL of dechlorinated tap water and the exact number of cercariae. After 45 min, they were returned to their cages. Residual cercariae were counted, and mice that received less than 95% of the cercariae were excluded from the experiment [21].

One hundred forty BALB/c mice were used in this preclinical trial. The animals were divided into seven groups, with 20 mice per group. The seven groups were as follows: group I was a positive control (infected with *S. mansoni* cercariae, but untreated), group II was a negative uninfected control, group III was pretreated with garlic (50 mg/kg) and infected with *S. mansoni* cercariae, group IV was pretreated with allicin (0.5 μM/mouse) and infected with *S. mansoni* cercariae, group V was infected with *S. mansoni *cercariae and treated with garlic (50 mg/kg), group VI was infected with *S. mansoni* cercariae and treated with allicin (0.5 μM/mouse), and group VII was infected and treated with PZQ at 500 mg/kg body weight. On day 49 post-infection with *S. mansoni*, group VII was treated with PZQ at 500 mg/kg in 70% glycerin on two successive days. Groups III and V received garlic (50 mg/kg) by oral gavage (single dose per day), and groups IV and VI received allicin (0.5 μM/mouse) by oral gavage (single dose per day). Daily administration of either garlic or allicin started 1 week before infection in groups III and IV or on the first day after infection in groups V and VI.

### Recovery of adult worms

On day 56 post-infection, 24 h after the final treatment, the mice were euthanized by decapitation. Blood samples were collected for serum analysis, and worms were recovered from the portal and mesenteric veins via vascular perfusion [22]. The perfused saline and blood drained from the portal vein were recovered in a beaker and left to form a sediment. The supernatant was removed, and the precipitate was washed twice with saline. After washing, the recovered worms were counted using a magnifying lens.

### Eggs per gram of liver

The number of *S. mansoni* eggs per gram of liver was counted as described elsewhere [23]. At the time of perfusion, entire mouse livers were weighed for all groups and a 0.5-g piece of liver was removed, placed into a screw cap glass tube, and frozen until digestion. At digestion, the frozen sample was crushed, 5 mL of 5% KOH was added to each tube, and the tubes were incubated at 37 °C until the tissue was completely digested (10–12 h). Egg counts from three 1-mL samples of the suspension were determined via microscopic examination at 40× magnification. One hundred eggs were randomly selected, microscopically examined, and classified as dead, immature, or mature for all groups.

### Intestinal egg counts

A 1 cm piece of terminal ileum was removed from each mouse. The intestine pieces were placed in petri dishes containing isotonic saline, opened end-to-end with scissors to remove the mucus, dried, weighed, placed between a glass slide and a plastic cover, and pressed on a rubber surface padded with absorbent paper [24, 25]. Samples were inspected under a light microscope at 100× magnification (or 400× in uncertain cases) and all eggs per slide were counted and classified by their developmental or maturation stage based on the specific features of each stage. A qualitative and quantitative oogram evaluation was performed, and in each intestinal fragment an average of 100 eggs were detected and classified as viable mature (containing a well-developed miracidium), immature first stage (embryo at one-third of the diameter of the egg), immature second stage (embryo at one-half of the length of the egg), immature third stage (embryo at two-thirds of the length of the egg), immature fourth stage (embryo almost entirely occupying the egg shell), or dead (calcified and semitransparent with a retracted miracidium) [24].

### Histopathological analysis and granuloma measurement

After portal perfusion, liver tissue pieces were fixed in 10% neutral formalin for 24 h, and paraffin blocks were prepared and processed for examination by light microscopy. Slices with a thickness of 250 μm were obtained from the prepared blocks and stained with hematoxylin, eosin, and Masson’s trichrome. Granuloma sizes were assessed in the histological sections [25], only those enclosing a single egg (with complete or collapsed miracidia), using an ocular micrometer. The preparations were viewed using a Nikon microscope at magnification × 400.

### Biochemical analysis

Serum samples were collected to assess the effects of garlic and allicin on mouse livers. Alanine aminotransferase (ALT) and aspartate aminotransferase (AST) concentrations were measured using commercial kits (Roche) on a Reflotron® Plussy system (Roche, Mannheim, Germany), and the results were statistically analysed to assess differences between samples before and after treatment.

### Immunohistochemical analysis

The standard avidin-biotin immunoperoxidase technique was used as described previously [26]. Paraffin samples (5 μm thick) were sectioned onto positively charged slides, deparaffinized in xylene, and hydrated in decreasing concentrations of ethanol. Endogenous peroxidase activity was reduced by incubation in 100% methanol with 3% hydrogen peroxide for 20 min. Antigen retrieval was performed by incubating the slices in citrate buffer (pH 7.0) and microwaving at 700 W for 15 min. The slices were incubated overnight at + 4 °C in a humidified chamber with primary monoclonal antibodies against mouse fibronectin and smooth muscle actin (SMA) alpha (Santa Cruz Biotechnology, Dallas, TX, USA). The antibodies were diluted 1:50 in phosphate-buffered saline (PBS). After one rinse in PBS, the slices were incubated at room temperature for 15 min with a biotinylated secondary anti-mouse antibody, washed again in PBS, and incubated with an avidin-biotin complex horseradish peroxidase solution (DAKO, Glostrup, Denmark). After 10 min of incubation, the peroxidase reaction was developed using 0.01% hydrogen peroxide in 0.05% diaminobenzidinetetrahydrochloride (DAB). The tissue slices were counterstained with Meyer’s haematoxylin and dehydrated in a graded series of ethanol solutions prior to mounting. Liver slices with the primary antibody replaced by PBS served as negative controls, while colonic cancer slices served as positive controls for fibronectin and SMA-alpha. The liver slices were examined under a Zeiss light microscope (Oberkochen, Germany). Fibronectin and α-SMA expression manifested as a brownish cytoplasmic staining in the hepatocytes, sinusoids, and collagen fibres of the granuloma.

### RNA extraction and reverse transcription

Total RNA was extracted using the Cultured Cell Total RNA Purification Mini Kit (Favorgen, Germany). After extraction, total RNA concentration and purity were evaluated using an Agilent 2100 Bio-analyzer system and an Agilent Small RNA analysis kit (Agilent technologies, Waldbronn, Germany). RNA (1 μg per sample) was reverse transcribed into cDNA using a high-capacity cDNA reverse transcription kit from Applied Biosystems (Warrington, USA) at 37 °C for 2 h.

### Real-time polymerase chain reaction (RT-PCR)

Quantitative PCR (qPCR) was conducted as previously described [27]. Briefly, the mRNA transcripts were measured using a PCR SYBR Green Supermix from Applied Biosystems with specific primers (Table 1). GAPDH served as the reference gene. The reaction was run in a 7500 Fast Real-Time PCR Thermal Cycler (Veriti ® 96- Well Thermal Cycler). The results were analysed using the 2-∆∆Ct (Livak) relative expression method.

**Table 1:** List of primers used for real-time PCR

Gene	Forward (F) and Reverse (R)
IL-13	F: CGGCAGCATGGTATGGAGTG
	R: ATTGCAATTGGAGATGTTGGTCAG
(tTG) Tissue	F: GTGAGCCGTGCTATCTGTCCTG
Transglutaminase	R: ACTGCCTGCTTGGAACCTGAA
IL-1β	F: GTGCTGGCTGGCCCACA
	R: GAACACCACTTCTCTCTTCA
IL-6	F: GGTACATCCTCGACGGCATCT
	R: GTGAAAGCAGCAAAGAGGCACT
TNF-α	F: AACCTCAGATAAGCCCGTCG
	R: ACCACCAGCTGGTTGTCTTT
GAPDH	F: TGTGTCCGTCGTGGATCTGA
	R: TTGCTGTTGAAGTCGCAGGAG

### Statistical analysis

Data are presented as the means and standard errors of the mean (SEM) or standard deviations using the Statistical Package for the Social Sciences (SPSS v. 22, Chicago, IL, USA). All statistical comparisons between the control and treated groups were made using one-way analysis of variance (ANOVA) followed by Dunnett’s post hoc test for multiple comparisons. Comparisons between groups were conducted using a Student’s t-test [28].

## Results

### Worm recovery

Analysis of the parasites at 56 days post-infection shows differences in the total number of recovered worms in the infected and treated mice compared to the positive control group. As shown in Table 2, statistical analysis shows that administering garlic and allicin significantly reduced the mean worm count 56 days after cercarial exposure (21.88% and 20.33%, respectively) compared to the positive control. PZQ-treated mice also showed a significant reduction (22.33%) compared to the positive control (Table 2). The treatment strategies significantly affected the oogram patterns compared to the positive control. Statistical analysis of the observed differences is presented in Table 2 and shows that administering garlic, allicin, or PZQ significantly reduced the total ova counts in the tissue (12.59%, 11.42%, and 19.32% respectively).

**Table 2:** Comparison between the positive control (no treatment) group and  the treated groups

Number of ova/gm/group		Positive Control	Prophylaxis with garlic	Prophylaxis with Allicin	Garlic	Allicin	Praziquantel
Mean Worm Burden ± SE	Male	7.5 ± 2.06	6.15 ± 2.1	6.44 ± 2.16	6.24 ± 2.13	6.59 ± 2.21	6.46 ± 2.09
	Female	2.8 ± 1.39	2.44 ± 1.20	2.40 ± 1.1	2.48 ± 1.17	2.55 ± 1.16	2.76 ± 1.25
	Couple	5.5 ± 0.97	3.92 ± 1.71*	3.83 ± 1.44*	4.02 ± 1.77*	3.98 ± 1.59*	3.66 ± 1.54**
	Total	21.3 ± 2.35	16.39 ± 5.69*	16.80 ± 5.13*	16.64 ± 5.69*	16.97 ± 5.28*	16.41 ± 4.89**
% Total Worm Burden Reduction			21.91***	20.15***	21.88***	20.33***	22.33***
Number of Ova/gm	Liver	3223.7 ± 732.45	2719.25 ± 820.2	2770.66 ± 817.85	2720.14 ± 822.22	2771.81 ± 818.97	2502.39 ± 867.65
	Intestine	3390.4 ± 741.58	3057.25 ± 871.8	3086.11 ± 871.69	3061.35 ± 877.77	3087.09 ± 871.69	2834.15 ± 908.98
% Reduction of Total Ova Count in Tissues			11.94***	11.39***	12.59***	11.42***	19.32***
% Egg Developmental Stages ± SE	%Immature	54.5 ± 1.58	48.26 ± 10. 81	46.45 ± 11.11	47.96 ± 11.18	46.48 ± 11.25	46.06 ± 10.99
	%Mature	39.4 ± 1.89	40.50 ± 8.97	40.38 ± 9.45	40.36 ± 9.07	40.52 ± 9.59	39.66 ± 9.42
	%Dead	6.1 ± 0.99	7.47 ± 2.57	7.88 ± 2.76	7.52 ± 2.71	8.01 ± 2.76	8.94 ± 3.29

Values are expressed as the mean number (M) ± standard deviation (SD)

^***^Highly significant statistical difference from infected control mice at *P* < 0.001

^**^Statistically significant difference from infected control mice at *P* < 0.01

^*^Statistically significant difference from infected control mice at *P* < 0.05

### Histopathological changes

**Fig. 1:**
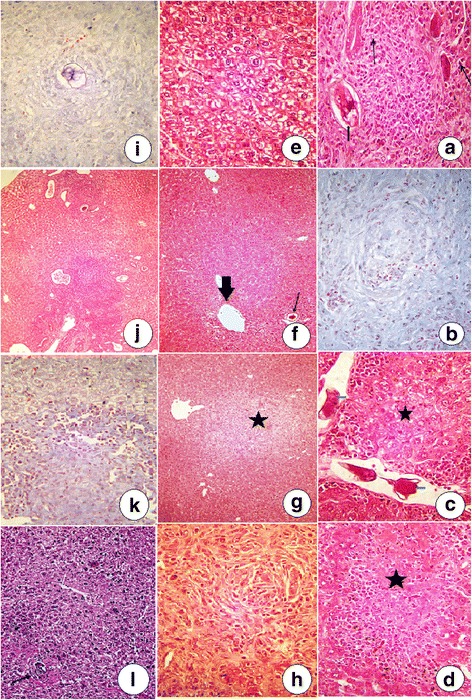
Light microscopy of mouse livers. **a** Group I showing a schistosome egg surrounded by cellular infiltrate (arrows) next to bile ductal proliferation (H&E, ×1200 magnification). **b** Group I showing extensive fibrosis of the hepatic parenchyma with collagen fibres stained blue by Masson’s Trichrome (×1200). **c** Group I showing numerous schistosome eggs inside the portal vein (arrows) surrounded by inflammatory reactions and acute swelling of hepatic cells (asterik). H&E staining (×1200). **d** Group I showing extensive coagulative necrosis (asterisk) infiltrated by eosinophils in the hepatic parenchyma. H&E staining (×1200). **e** Group II showing apparently normal hepatic parenchyma. H&E staining (×1200). **f** Group III showing a few eggs with granuloma (arrow) surrounded by small granulomatous reactions with apparently normal hepatic parenchyma and small portal inflammatory reactions (thick arrow). H&E staining (×300). **g** Group IV showing a small granuloma (without an egg) containing minimal collagen deposits followed by normal hepatic parenchyma without inflammatory reactions in portal areas. H&E staining (×300). **h** A higher-power version of the Fig. 1g showing minimal collagen fibres and inflammatory cells without an egg in the granuloma. H&E staining (×1200). **i**) Group IV showing a degenerated egg surrounded by mild collagen fibres stained blue by Masson’s trichrome (×1200). **j** Group V showing scattered egg granulomata and inflammatory reactions in some portal areas. H&E staining (×300). **k** Group V showing moderate quantities of collagen fibres stained blue in the granuloma by Masson’s Trichrome (×1200). **l** Group VI showing small fibrocellular granulomata formed around a central dead egg and surrounded by lymphocytes, histiocytes, fibroblasts, and concentric collagen fibres (black arrow; H&E staining, ×200)

In group I, numerous schistosome eggs were surrounded by an intense granulomatous reaction from macrophages, eosinophils, fibroblasts, and lymphocytes that included giant cells (Fig. 1a). Additionally, most of the hepatic parenchyma were replaced. Most granulomata had extensive fibrosis, and the collagen fiber was stained blue by Masson’s trichrome staining (Fig. 1b). The portal area showed numerous eggs within the portal vein and portal vessel walls, and the adjacent area contained active proinflammatory cells, mainly eosinophils and lymphocytes (Fig. 1c). These occasionally formed lymphoid nodules in the portal area or interstitial tissue, or the adjacent parenchyma, developed extensive acute cell swelling or coagulative necrosis, usually infiltrated by eosinophils (Fig. 1d). Most of the hepatic cells displayed karyomegaly adjacent to hyperplastic Kupffer cells in the presence of scattered bilharzial pigment in the hepatic parenchyma. In group II, all hepatic parenchyma was apparently normal (Fig. 1e). Group III showed few egg granulomata containing degenerated eggs surrounded by small granulomatous reactions from lymphocytes. Macrophages without giant cells or with few portal inflammatory reactions but apparently normal hepatic parenchyma were common (Fig. 1f). Most granulomata were small and contained degenerated eggs surrounding small amounts of fibrosis and collagen fibres stained blue by Masson’s trichrome (Fig. 1g). A few collagen fibres infiltrated by eosinophils were observed in subcapsular areas, and a few apoptotic bodies and acutely swollen cells were observed in the adjacent hepatic parenchyma. Some portal areas exhibited mild vacuities in some of the portal vessels. Small scattered granulomata without eggs, containing minimal collagen deposits and accompanied by normal hepatic parenchyma without inflammatory reactions in portal areas were common in group IV (Fig. 1h). The granulomata had minimal collagen fibres, which were usually infiltrated by eosinophils (Fig. 1i). The eggs were usually degenerated and calcified with distorted shells and no embryos. Most of the surrounding parenchyma was normal except for a few scattered cells that showed acute swelling. Most hepatic parenchyma appeared normal, except for several scattered egg granulomata consisting of a few eggs surrounded by inflammatory reactions mediated by eosinophils, lymphocytes, and fibroblasts. Moderate collagen deposits replaced most of the granulomata in group V (Fig. 1j, k). Liver slices from group VI showed small fibrocellular granulomata consisting of a central dead egg surrounded by lymphocytes, histiocytes, fibroblasts, and concentric collagen fibres (Fig. 1l).

### Biochemical analysis: Effects of garlic and allicin on ALT and AST synthesis

**Fig. 2:**
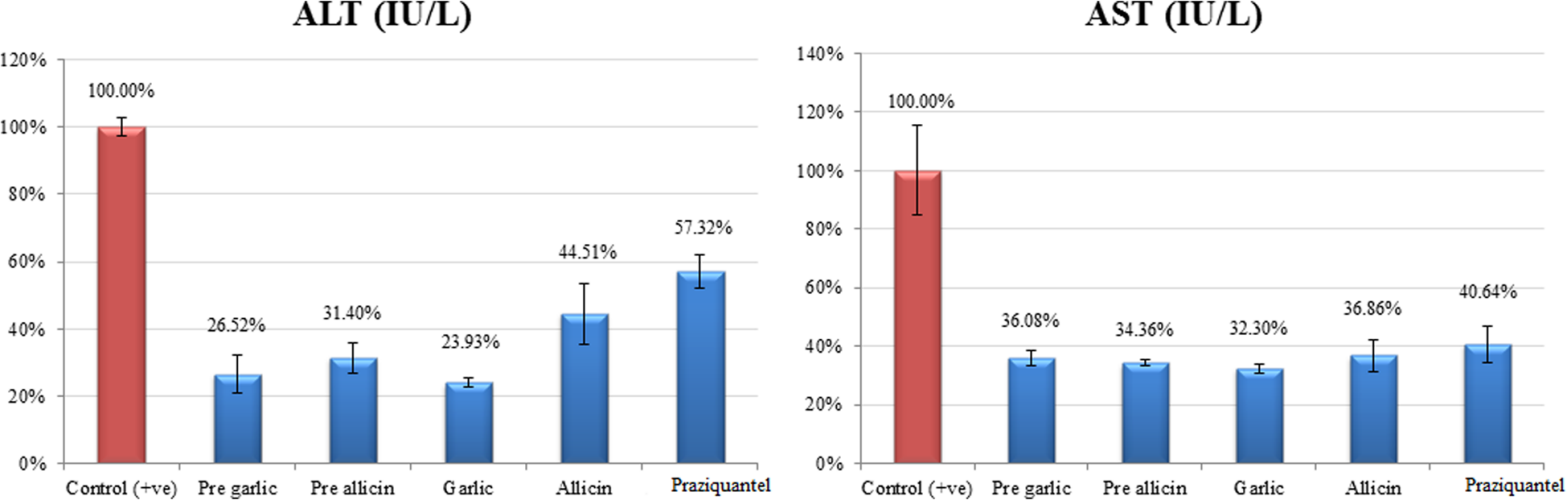
Percentage change in ALT (IU/L) and AST (IU/L) in all groups compared to positive control

Data are presented as the mean ± SD of at least 20 independent measurements. Figure 2 shows the percentage change due to prophylaxis with garlic, prophylaxis with allicin, treatment with garlic, treatment with allicin, and treatment with PZQ relative to the infected non-treated control (Table 3) and (Fig. 2).

**Table 3:** Determination of serum biochemical parameters

Parameter	Group	N	Min.	Max.	Mean ± S.D.	*P* value^a^	*P* value^b^
ALT (IU/L)	Positive Control	20	160.00	170.00	164.00 ± 4.32		0.001^*^
	Pre-garlic	20	33.00	55.00	43.50 ± 9.33	0.001^*^	
	Pre-allicin	20	44.00	60.00	51.50 ± 7.72	0.001^*^	
	Garlic	20	37.00	42.00	39.25 ± 2.22	0.001^*^	
	Allicin	20	53.00	88.00	73.00 ± 14.58	0.001^*^	
	Praziquantel	20	88.00	106.00	94.00 ± 8.16	0.001^*^	
AST (IU/L)	Positive Control	20	251.00	354.00	291.00 ± 44.77		0.001^*^
	Pre-garlic	20	98.00	115.00	105.00 ± 7.26	0.001^*^	
	Pre-allicin	20	97.00	103.00	100.00 ± 2.58	0.001^*^	
	Garlic	20	90.00	101.00	94.00 ± 4.83	0.001^*^	
	Allicin	20	92.00	123.00	107.25 ± 16.01	0.001^*^	
	Praziquantel	20	101.00	138.00	118.25 ± 18.06	0.001^*^	

^a^*P* value between positive control group and the other groups using Dunnett’s test for multiple comparisons

^b^*P* value among all groups using one-way ANOVA

^*^Indicates a significant difference between the group and the positive control

### Effects of garlic and allicin on fibrosis biomarkers

**Fig. 3:**
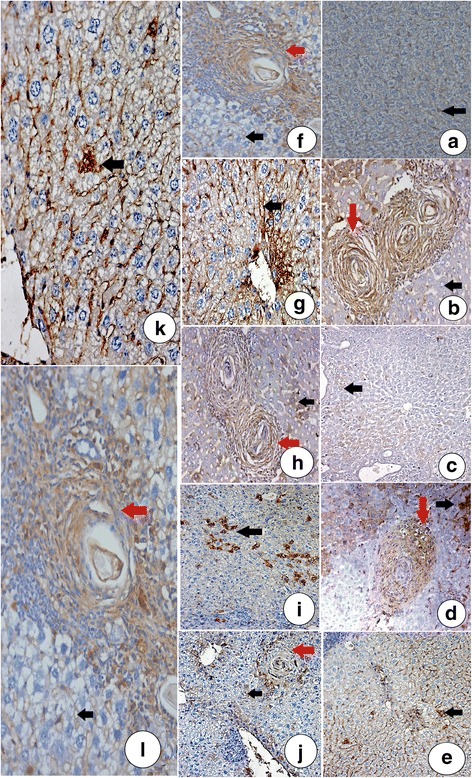
Immunohistochemical staining for fibronectin and α-smooth muscle actin (IHC, DAB, ×200). Liver sections from: **a** Group II showing positive expression of fibronectin in the sinusoids as brownish material (black arrow). **b** Group I with *S. mansoni* showing a large fibrocellular bilharzial granuloma positive for fibronectin, and brownish stained collagen bundles (red arrow). Hepatocytes are also shown (black arrow). **c** Group III showing hepatic tissues positive for fibronectin, stained brownish (black arrow). **d** Group IV showing small fibrocellular granulomata, positive for fibronectin immunostaining in both granuloma (red arrow) and hepatocytes (black arrow). **e** Group V showing few hepatocytes positive for fibronectin immunostaining (black arrow). **f** Group VII with small fibrocellular granulomata, positive for fibronectin in both the granuloma (red arrow) and hepatocytes (black arrow). **g** Group II showing positive expression of α-smooth muscle actin in the sinusoids as brownish material (black arrow). **h** Group II showing a large fibrocellular bilharzial granuloma positive for α-smooth muscle actin. Collagen bundles are stained brownish (red arrow) and hepatocytes are also indicated (black). **i** Group III showing few hepatic cells positive for α-smooth muscle actin, stained brownish (black arrow). **j** Group IV showing small fibrocellular granulomata positive for α-smooth muscle actin in granuloma (red arrow). Hepatocytes are also shown (black arrow). **k** Group V showing few hepatocytes positive for α-smooth muscle actin (black arrow). **l** Group VII showing small fibrocellular granulomata positive for α-smooth muscle actin in the granuloma (red arrow). Hepatocytes are also shown (black arrow)

As shown in Fig. 3, the sinusoids in the normal mouse liver stained positive for fibronectin and α-SMA. Groups III, IV, V, and VI showed similarly distributed and decreased fibronectin and α-SMA cells. There was a notably large number of both fibronectin and α-SMA-positive cells in group I that showed large fibrocellular bilharzial granuloma with brownish stained collagen bundles (Fig. 3).

### Anti-inflammatory activity of garlic and allicin

**Fig. 4:**
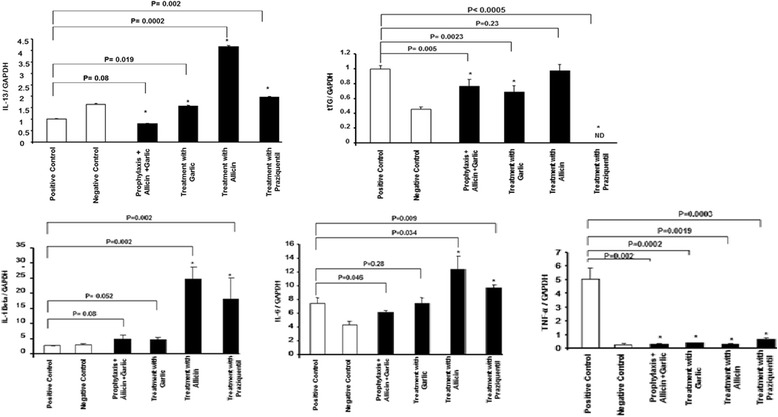
Relative cytokine mRNA expression levels after infection with *S. mansoni* cercariae and different therapeutic treatments

We analysed proinflammatory cytokine expression in the liver after various treatments. Figure 4 shows the impact of garlic, allicin, and PZQ on the expression levels of IL-13, tTG, IL-1β, IL-6, and TNF-α. The parasite had no effect on IL-13 expression. Allicin and garlic treatment increased IL-13 expression. However, exposing the mice to garlic prophylactically decreased the IL-13 gene expression. tTG expression increased during infection. Allicin and garlic treatment reduced, and PZQ treatment eliminated, tTG gene expression (not detected, ND). IL-1β and IL-6 expression increased after both prophylaxis and treatment. TNF-α expression also increased after infection with cercariae (infected no-treatment control). In contrast, TNF-α expression decreased in the prophylaxis and treatment group tissues.

## Discussion

Liver fibrosis due to *S. mansoni* is a severe pathological change that promotes loss of liver function. The disease results in a hepatic immune and inflammatory reaction with varying degrees of progression to cirrhosis. No treatment has been shown to directly affect the parasite. Therefore, dependable methods to decelerate or eliminate liver fibrosis are needed. Although a chemical drug reduces adult worm burden and inhibits schistosome egg accumulation, less direct treatments aim to diminish hepatic fibrosis, primarily during the chronic stages of schistosomiasis. Thus, developing a treatment to target schistosomiasis induced hepatic fibrosis remains a challenge [29]. Garlic (*Allium sativum*) has antischistosomal effects, as it upregulates nitric oxide (NO) production in blood platelets and macrophages, that destroy the parasite [30]. Garlic also contains mannose-binding lectins [31], which facilitate attachment of the parasite to the macrophage surface receptor, allowing the macrophage to engulf the parasite [32]. In addition, garlic contains an immunomodulatory fraction, which shifts the cytokine pattern from T helper 2 (Th2)-lymphocyte-mediated immune responses, responsible for granuloma formation. to Th1-lymphocyte-mediated immune responses responsible for immune resistance [32].

Murine liver fibrosis was induced by *S. mansoni* cercariae infection, which was treated with crushed garlic homogenate, allicin, or PZQ. At 56 days post-infection, the total number of recovered worms and eggs decreased significantly in the garlic and prophylactic treated mice compared to the positive control. The reduced egg count may be attributed to a reduced worm burden and/or the drugs may affect the ability of the worms to copulate, thereby affecting egg production by the female adult worms. This study suggests an anti-inflammatory mechanism behind the antischistosomal action of garlic and allicin rather than a direct effect on the parasites. These results are consistent with other studies [16, 32–37].

The localization and concentration of certain marker enzymes for various cell organelles are affected by hepatic cells, and defects in these enzymes will affect enzymatic activity [36]. Therefore, analysing changes in enzyme activity helps to assess the probable adverse effects of different treatments on various cell organelles. Serum ALT and AST activities are biomarkers for hepatic cell damage caused by heavy schistosome egg deposition [37]. In this study, ALT and AST activities increased progressively in *S. mansoni*-infected mice compared to the prophylactic and treated groups due to greater hepatic damage. Once it begins, fibrosis accelerates the inflammatory necrosis process via cytokines. Garlic extract protects the hepatic cells, decreasing serum ALT and AST levels in mice compared to the positive control [38].

Garlic administration exerted marked anti-inflammatory action, decreasing granuloma size as well as the number of collagen fibres, inflammatory cells, and eggs in the granuloma compared to the positive control.

The administration of garlic and allicin was comparable with that of the standard drug PZQ. PZQ effects included small fibrocellular granulomata consisting of a central dead egg surrounded by lymphocytes, histiocytes, fibroblasts, and concentric collagen fibres. Garlic administration resulted in marked anti-inflammatory activities, because it significantly reduced granuloma volume [15]. Accordingly, the infiltration of circulating fibroblasts into the granulomata may be necessary to attract lymphocytes and form collagen, indicating that garlic’s fibrinolytic effect may decrease the diameter and cellularity of the granuloma [39, 40] due to its antioxidant properties [17].

After liver damage, hepatic stellate cells (HSCs) undergo a complex transformation or activation process in which the cells change from a quiescent, vitamin A-storing, cell type into activated myofibroblasts. This transformation results in considerable changes including the altered appearance of the cytoskeletal protein smooth muscle α-actin (α-SMA), loss of cellular vitamin A stores [41], and upregulation of type I and III collagen genes. These pathogenic changes cause excessive deposition of ECM proteins including three large families of glycoproteins, collagens, proteoglycans [42], and fibronectin [43] that disrupt the balance of ECM integrity and thereby induce hepatic fibrosis. In this study, immunohistochemical analysis of fibronectin and α-SMA was conducted because they are sensitive markers whose levels increase significantly during liver fibrosis [34, 44].

Both markers were upregulated in the infected untreated groups. However, their expression decreased significantly when garlic, allicin, and PZQ were administered. These data suggest anti-inflammatory and immunomodulatory effects from the garlic, allicin, and PZQ.

These data are consistent with experimental studies demonstrating that activated HSCs participate in ECM deposition in periovular schistosomal granulomata [45–48].

Cytokines modulate the extent of fibrosis and the granuloma size and are important in schistosomal infection pathogenesis [49]. As a critical profibrotic cytokine found in various organs, including the liver, IL-13 is thought to be the key mediator of liver fibrosis in *S. mansoni* infections [50, 51]. IL-1 and TNF-α participate in maintaining the granulomatous response [52]. Tissue transglutaminases (tTGs), a group of multifunctional enzymes, are central in the pathogenesis of chronic liver diseases, [53] and are important inflammatory and fibrotic factors involved in Th2 responses [54]. tTGs are associated with cytokines such as IL-6 [55] and IL-13 [56]. By activating a Th2 response, tTGs may enhance IL-6 [55] and IL-13 production [56]. Moreover, expression of Th1 or Th2 cytokines important for liver fibrosis, such as IL-6, IL-10, IL-13, and TNF-α [57], were also detected in our experiment. These data indicate that fibrotic development requires the production of the profibrotic cytokines IL-6 and TNF-α. In contrast, IL-13 and IL-1β showed no profibrotic effects in our model. Garlic’s effect was larger than that of PZQ. Antifibrotic treatment with garlic significantly inhibited inflammatory cytokine expression, suggesting that the altered Th1/Th2 cytokine balance after garlic treatment may eventually facilitate the resolution of hepatic fibrosis. This result is consistent with another report, [54] in which investigators studied the relationship of IL-13, tTG, liver granuloma, and fibrosis after *S. japonicum* infection.

## Conclusion

In this study, immunohistochemical expression of fibronectin and α-SMA, as well as mRNA expression of inflammatory cytokines serving as markers of hepatic fibrosis, reflected significant anti-inflammatory and immunomodulatory effects after both prophylactic administration and treatment of infected mice with garlic extract or allicin. This suggests that these substances are promising adjunctive therapeutics for schistosomiasis. Further molecular studies are recommended to examine the effects of garlic and allicin on the apoptotic pathway.

## Abbreviations

ALT: Alanine aminotransferase
AST: Aspartate aminotransferase
BALB/c: Bagg albino, laboratory-bred mouse strain
cDNA: complementary DNA
cDNA: complementary DNA
DAB: Diaminobenzidinetetrahydrochloride
ECM: Extracellular matrix
GAPDH: Glyceraldehyde-3-phosphate dehydrogenase
HSCs: Hepatic stellate cells
IL: Interleukin
KOH: Potassium hydroxide formula
mRNA: messenger RNA
ND: Not detected
NO: Nitric oxide
PBS: phosphate-buffered saline
PZQ: Praziquantel
RNA: ribonucleic acid
RT-PCR: Real-time polymerase chain reaction
TBRI: Theodor Bilharz Institute
Th1: T helper type 1
Th2: T helper type 2
TNF-α: tumour necrosis factor alpha
tTGs: tissue transglutaminases
α-SMA: Alpha-smooth muscle actin
